# Death of Dr. Robert Egglesfeld Griffith

**Published:** 1850-07

**Authors:** 


Died, in Philadelphia, Dr. Robert Egglesfeld Griffith, in the 53d year of his age.

Dr. Griffith was essentially one of the working men of the profession. Although in feeble health for some years past, his active mind was constantly engaged in adding to the stores of medical literature. Among the productions of his pen we may mention the Universal Formulary, of which we recently gave a notice, a work on Medical Botany, an American edition of Taylor's Medical Jurisprudence, and Christison's Materia Medica. To his friends his loss will be irreparable, and the void created by his death will be hard to fill.

